# Automated pancreas segmentation and volumetry using deep neural network on computed tomography

**DOI:** 10.1038/s41598-022-07848-3

**Published:** 2022-03-08

**Authors:** Sang-Heon Lim, Young Jae Kim, Yeon-Ho Park, Doojin Kim, Kwang Gi Kim, Doo-Ho Lee

**Affiliations:** 1 Department of Health Sciences and Technology, Gachon Advanced Institute for Health Sciences and Technology (GAIHST), Gachon University, 1342, Seongnamdaero, Sujeong-gu, Seongnam-si, 13120 Republic of Korea; 2 Department of Biomedical Engineering, Gil Medical Center, Gachon University College of Medicine, 38-13, Dokjeon-ro 3beon-gil, Namdong-gu, Incheon, 21565 Republic of Korea; 3 Department of Surgery, Gil Medical Center, Gachon University College of Medicine, 774-21, Namdong-daero, Namdong-gu, Incheon, 21565 Republic of Korea

**Keywords:** Anatomy, Health care, Engineering, Mathematics and computing

## Abstract

Pancreas segmentation is necessary for observing lesions, analyzing anatomical structures, and predicting patient prognosis. Therefore, various studies have designed segmentation models based on convolutional neural networks for pancreas segmentation. However, the deep learning approach is limited by a lack of data, and studies conducted on a large computed tomography dataset are scarce. Therefore, this study aims to perform deep-learning-based semantic segmentation on 1006 participants and evaluate the automatic segmentation performance of the pancreas via four individual three-dimensional segmentation networks. In this study, we performed internal validation with 1,006 patients and external validation using the cancer imaging archive pancreas dataset. We obtained mean precision, recall, and dice similarity coefficients of 0.869, 0.842, and 0.842, respectively, for internal validation via a relevant approach among the four deep learning networks. Using the external dataset, the deep learning network achieved mean precision, recall, and dice similarity coefficients of 0.779, 0.749, and 0.735, respectively. We expect that generalized deep-learning-based systems can assist clinical decisions by providing accurate pancreatic segmentation and quantitative information of the pancreas for abdominal computed tomography.

## Introduction

The detection rate of benign or malignant lesions of the pancreas, and subsequent surgery are gradually increasing, owing to the early diagnosis of pancreatic neoplasm, which is a result of the development of imaging modalities, an increase in the health screening program, and the aging of the population^1–4^. In particular, cystic tumors that are inadvertently identified in the pancreas require continuous follow-up^2, 3^. This is typically followed by computed tomography (CT) scans of the abdomen to observe the increase in lesion size. Subsequently, resection of benign or malignant tumors on the endocrine and exocrine function of the pancreas is implemented in the long-term, which greatly affects the patient’s quality of life^4–6^.

It is necessary to investigate the change in the volume of the pancreas after resection; however, this is difficult to apply in clinical practice because it is cumbersome and laborious to obtain the volume of the pancreas from abdominal CT using current technology^5, 6^. The quantitative pancreatic volume cannot be measured in all patients after resection of the pancreas because obtaining the volume of the pancreas is a long and time-consuming task. In addition, determining the volume of the pancreas by hand is error-prone for each examiner. Therefore, this situation necessitates a computer-aided diagnosis (CAD) system based on artificial intelligence.

Automatically obtaining the volume of the pancreas from abdominal CT scans based on artificial intelligence can assist in calculating the quantitative pancreatic volume and the patient’s endocrine and exocrine functions, which enables a more scientific and objective treatment for the patients. Therefore, the current study develops a technique for calculating the volume of the pancreas based on deep learning technology using abdominal CT scan images.

Recently, deep learning (DL)-based semantic segmentation networks were considered more beneficial for medical image segmentation tasks compared with traditional image segmentation methods, such as the intensity-based threshold, morphology, and geometry^7–10^. However, accurate pancreas segmentation is a challenging task because the pancreas is structurally diverse, it occupies a small region in the abdomen, and it is closely attached to other organs, such as the duodenum and gallbladder^11, 12^.

However, a convolutional neural network (CNN)-based method was proposed as a promising method for pancreas segmentation, owing to the powerful advantages of the DL method. Subsequently, several studies proposed state-of-the-art CNN-based pancreas segmentation approaches via either cascaded or coarse-to-fine segmentation networks. However, previous pancreas segmentation studies were performed on small study populations^11, 13, 14^ that comprised 82 participants from the National Institutes of Health (NIH) clinical center. Furthermore, DL methods are sensitive to the features of the data that are encoded in the network; therefore, clinical assessment of the pancreatic segmentation performance is necessary for varied and large datasets. However, to the best of our knowledge, DL studies on large CT datasets that contain various pancreatic volumes are scarce.

Therefore, we aim to evaluate the performance of four DL-based three-dimensional (3D) pancreas segmentation networks on 1006 healthy participants. In addition, we evaluate the reliability of the pancreatic-volume estimation task using DL-based approaches. In this study, we exploit four semantic segmentation networks based on a 3D u-net. One of the four networks is the basic 3D u-net, and the other three networks are configured with residual modules, dense modules, and residual dense modules. We assess DL networks using segmentation metrics (i.e., dice similarity coefficient, precision, and recall) as well as a regression plot and Bland–Altman plot for pancreatic volumetric evaluation.

## Methods

### Study populations

We acquired abdominal CT images from 1006 patients, who were examined at the Gil Medical Center. All patient records were confirmed and retrospectively reviewed based on a clinical diagnosis from 2016 to 2019. This study was conducted in accordance with the Declaration of Helsinki and written informed consent was obtained from all the participants (IRB number: GDIRB2020-121). This study was approved by the Institutional Review Board of the Gil Medical Center. The inclusion criteria for this study were as follows: (1) patient did not undergo pancreatic resection, and (2) patient has no benign or malignant tumor in the pancreas. The feature of the CT dataset has a slice thickness of 3–5 mm and a pixel spacing of 0.58–0.97 mm. We used a CT scanner (SOMATOM Definition Edge, Siemens, Germany), and images were acquired using a tube-voltage of 80–150 kVp and tube-current of 52–641 mA.

### Preprocessing and experimental setup

Manual delineation was conducted in the 2D axial plane using ImageJ (ver. 1.52a, NIH, USA) to generate a gold standard. As the acquired CT volumes have a different voxel-spacing couple, we unified the voxel spacing of all the volume data; the slice thickness was regularized to 3 mm, and the pixel spacing to 1 mm ($\mathrm{z},\mathrm{ y},\mathrm{ x}=3, 1, 1$). Moreover, because the manual delineation was conducted before conducting volume reconstruction, we simultaneously reconstructed the CT and mask volumes. Based on the reconstructed mask volume, a specific margin was assigned to crop the region of the pancreas.

**Figure 1:**
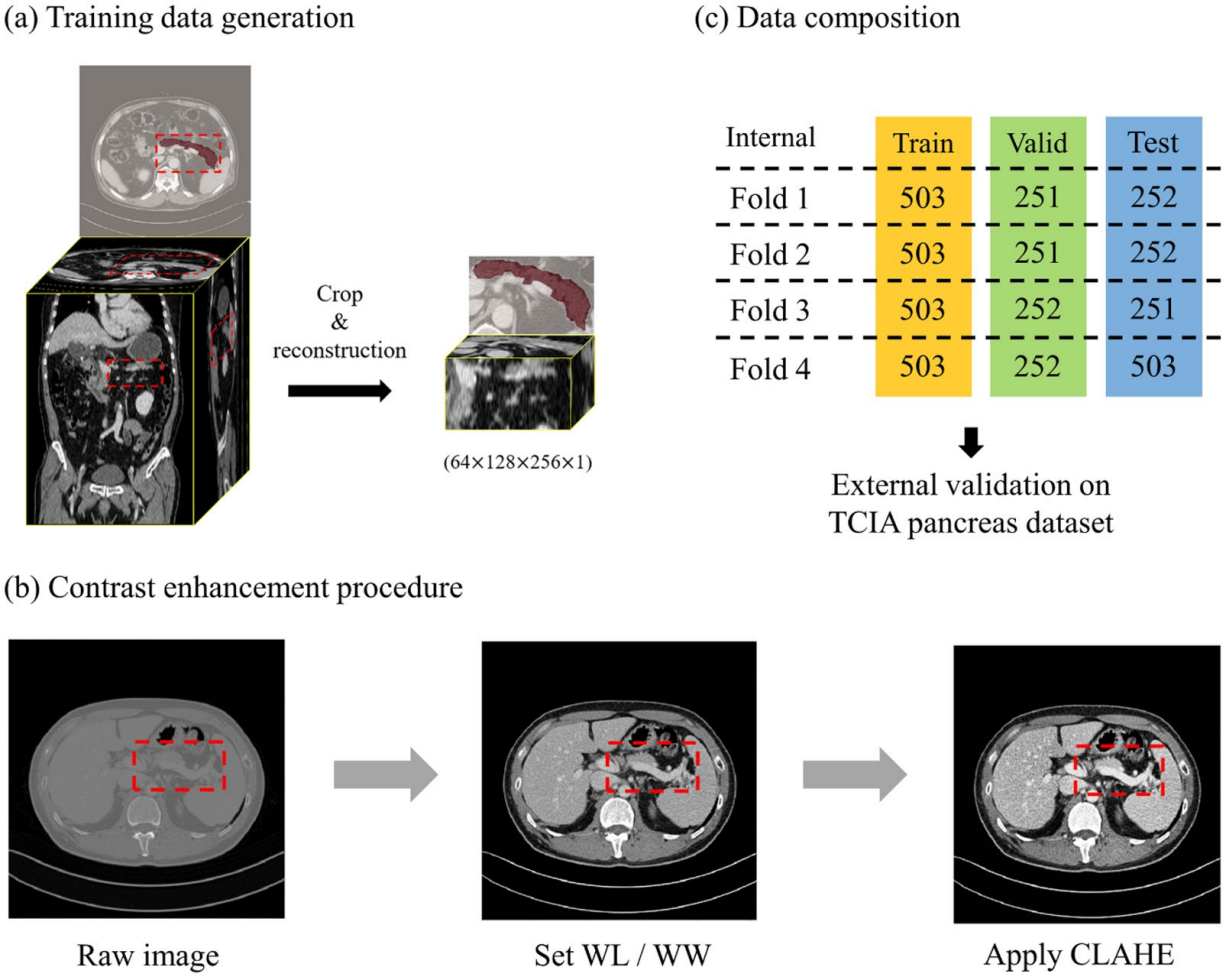
(**a**) Raw volume data were cropped and reconstructed for training data generation. (**b**) All data were divided into datasets that consisted of almost identical numbers of participants for cross validation. (**c**) A region of the pancreas was enhanced via CLAHE. Valid, validation; TCIA, the Cancer Imaging Archive; WL, window level; WW, window width; CLAHE, contrast-limited adaptive histogram equalization.

Owing to the irregular shape of the pancreas (x-, y-, z-axis), we cropped the image considering the ratio of the depth, width, and height of the pancreas (z:y:x = 1:2:3). Additionally, the volume of the cropped pancreatic region varies according to the patient; therefore, bilinear interpolation was applied to create a volume with a particular single-channel size (64, 128, 256, 1) (Fig. 1a). The pancreas cropping process was conducted based on manual delineation.

The pancreas is attached to other organs, such as the duodenum and gallbladder; therefore, contrast enhancement was applied to the input volume to increase the visibility of the pancreas. First, we adjusted the CT images using a window center (60) and window width (400) to clearly observe the region of the abdomen^15^. The final dataset was generated by applying contrast-limited adaptive histogram equalization (CLAHE) to enhance the contrast of the pancreatic region (Fig. 1b)^16–19^.

The output images and resized images were restored using the same voxel spacing as the raw CT data during preprocessing, before input to the network, to evaluate the pancreatic segmentation performance of the network. Additionally, we conducted fourfold cross validation on binary images that restored the voxel spacing of raw CT data (Fig. 1c). External validation was performed using the Cancer Imaging Archive (TCIA) pancreas-CT dataset^14, 20, 21^, which was provided by the NIH clinical center (n = 82). The TCIA dataset was split as the ratio of 10:5:5 for fourfold cross validation. The segmentation performance assessment was performed via a pixel-wise comparison between the gold standard and prediction results of the DL network (if probability > 0.5, positive). As a result of the assessment, we obtained a confusion matrix (i.e., true positive, false positive, true negative, and false negative) from 3D binary volume images.

### Network architecture

**Figure 2:**
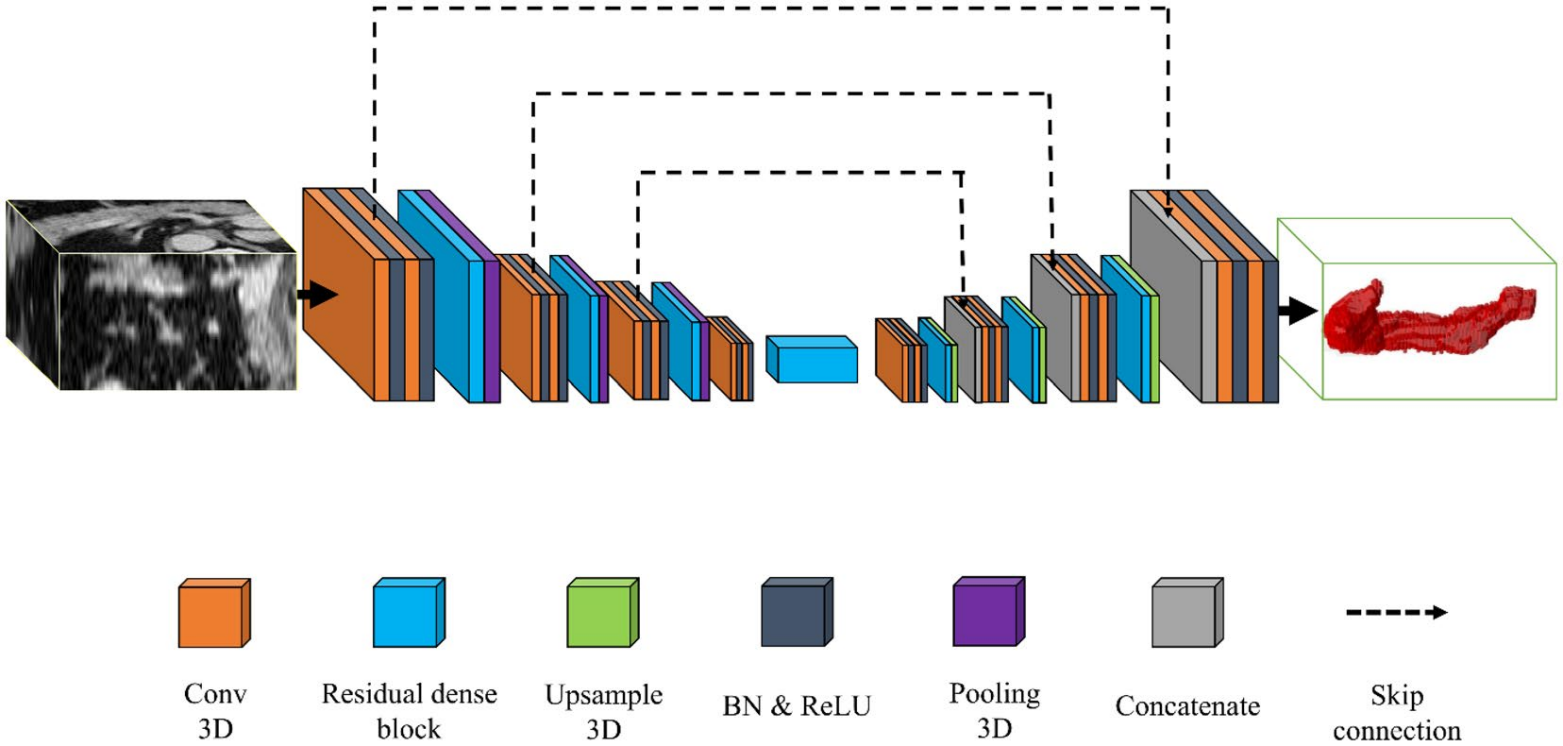
Architecture illustration of residual dense u-net. Conv, convolution; BN, batch normalization; ReLU, rectified linear unit.

We exploited 3D u-net-based architectures with skip connections and batch normalization, which consisted of four resolution steps^22–26^. All convolution blocks comprised a convolutional kernel size of ($3\times 3\times 3$), dilation rate^27^ of ($1\times 1\times 1$), and rectified linear units (ReLUs). The hyper-parameter setting was set to the hyper-parameter that achieved the best performance in all baseline networks. In addition, we employed simple 3D upsample layers instead of transposed convolution layers for the decoding steps. Figure 2 shows the architecture of the residual dense u-net for pancreas segmentation^28, 29^. We performed deep learning analysis using four semantic segmentation networks that had the same width, depth, and filter size, except for specific blocks (i.e., dense blocks, residual blocks, and residual dense blocks). For a network comparison, we experimented by replacing the blocks in the residual dense blocks in Fig. 2 with the other specific modules.

### Implementation details

This study conducted a deep learning analysis on a Tesla V100 (32 GB) graphics processing unit (GPU). The networks were trained using the Adam optimizer (learning rate 0.001) to minimize dice loss. We utilized the following frameworks using Python (ver. 3.6.12, Python Software Foundation, USA): Keras (ver. 2.2.5), TensorFlow-GPU (ver. 1.15.4). The training settings of all networks are as follows: batch size, 2; epoch, 500.

## Results

### Participant demographics

Table 1 shows the demographics of the participants who underwent abdominal CT for routine health check-ups via a health-care program. A total of 528 (52.6%) participants were men and 475 (47.4%) were women. The mean age was 55.3 years, and the mean body mass index was 24.3 kg/m^2^. Significant differences were observed in height (men: 169.3 ± 6.8 vs. women: 157.1 ± 6.2 cm, *p* < 0.001), weight (70.3 ± 11.6 vs. 59.6 ± 10.0 kg, *p* < 0.001), smoking (n = 224, 22.3% vs. n = 39, 8.2%, *p* < 0.001), and alcohol consumption (n = 246, 46.6% vs. n = 84, 17.7%, *p* < 0.001) between men and women. The mean volume of the pancreas was 66.5 cm^3^, and a significant difference was observed between men and women (68.8 ± 19.5 vs. 55.8 ± 16.0, *p* < 0.001). No significant differences were observed in men and women based on age, body mass index, and the proportion of those with hypertension and diabetes mellitus.

**Table 1:** Demographics of participants.

	Total	Men	Women	*p* value
Number	1006 (100)	530 (52.6)	476 (47.4)	
Age (years)	55.3 ± 15.6	55.6 ± 15.3	54.9 ± 15.9	0.508
Height (cm)	163.5 ± 8.9	169.3 ± 6.8	157.1 ± 6.2	< 0.001
Weight (kg)	65.3 ± 12.1	70.3 ± 11.6	59.6 ± 10.0	< 0.001
Body mass index (kg/m^2^)	24.3 ± 3.5	24.4 ± 3.3	24.1 ± 3.7	0.165
Hypertension	364 (36.3)	205 (38.8)	159 (33.5)	0.078
Diabetes mellitus	190 (18.9)	108 (20.5)	82 (17.3)	0.198
Smoking	224 (22.3)	185 (35.0)	39 (8.2)	< 0.001
Alcohol	330 (32.9)	246 (46.6)	84 (17.7)	< 0.001
Volume of pancreas (cm^3^)	62.6 ± 19.0	68.8 ± 19.5	55.8 ± 16.0	< 0.001

Values are expressed as *n* (%) or mean ± standard deviation, unless otherwise indicated.

### Pancreas segmentation

Table 2 presents the evaluation results of the four 3D segmentation models. Networks using residual dense blocks achieved the highest precision, recall, and DSC, and they also exhibited the lowest standard deviation. Additionally, we performed paired *t* tests to verify the statistical significance between residual dense u-nets and other networks. The results showed that the residual dense u-net was promising and significantly different from the other three networks (all significance levels were *p* < 0.05). In contrast, the residual u-net achieved the lowest pancreas segmentation performance in terms of precision, recall, and DSC. The residual dense u-net obtained a mean precision, recall, and DSC of 0.779 ± 0.204, 0.749 ± 0.226, and 0.735 ± 0.197, respectively, on the NIH external dataset. Furthermore, the residual dense u-net achieved the highest mean DSC for every pancreas volume range: (1) 0–30 cm^3^, mean DSC of 0.808; (2) 30–60 cm^3^, mean DSC of 0.851; (3) 60–90 cm^3^, mean DSC of 0.872; (4) > 90 cm^3^, mean DSC of 0.870 on our dataset (Table 3). The statistical assessment was performed on 2D axial plane.

**Table 2:** Evaluation metrics for four pancreas segmentation models.

	Precision	Recall	DSC	Trainable parameter
Basic U-net	*0.861 *$\pm$* 0.468*	*0.816 *$\pm$* 0.173*	*0.822 *$\pm$* 0.143*	11,003,073
Dense U-net	*0.864 *$\pm$* 0.114*	*0.828 *$\pm$* 0.165*	*0.831 *$\pm$* 0.134*	35,261,601
Residual U-net	*0.843 *$\pm$* 0.127*	*0.810 *$\pm$* 0.178*	*0.808 *$\pm$* 0.146*	2,350,857
Residual Dense U-net	**0.869 **$\pm$** 0.110**	**0.842 **$\pm$** 0.156**	**0.842 **$\pm$** 0.128**	47,074,657

Results are indicated as mean ± standard deviation, and the best performances are indicated in bold. The results are highlighted in italics if the residual dense u-net performs significantly better than the corresponding method. We used a significance level of 0.05 and a paired *t* test for network comparison.

DSC, dice similarity coefficient.

**Table 3:** Comparison of pancreas segmentation performance according to pancreatic volumes using four independent 3D networks.

	DSC	*P-value
PV $<$ 30 cm^3^ (n = 54)		
Basic U-net	0.785 $\pm$ 0.100	< 0.001
Dense U-net	0.794 $\pm$ 0.089	0.013
Residual U-net	0.756 $\pm$ 0.111	< 0.001
Residual Dense U-net	**0.808 **$\pm$** 0.078**	–
30 cm^3^ $\le$ PV $<$ 60 cm^3^ (n = 361)		
Basic U-net	0.834 $\pm$ 0.073	< 0.001
Dense U-net	0.842 $\pm$ 0.066	< 0.001
Residual U-net	0.815 $\pm$ 0.082	< 0.001
Residual Dense U-net	**0.851 **$\pm$** 0.060**	**–**
60 cm^3^ $\le$ PV $<$ 90 cm^3^ (n = 441)		
Basic U-net	0.859 $\pm$ 0.047	< 0.001
Dense U-net	0.866 $\pm$ 0.039	< 0.001
Residual U-net	0.844 $\pm$ 0.053	< 0.001
Residual Dense U-net	**0.872 **$\pm$** 0.037**	**–**
PV $\ge$ 90 cm^3^ (n = 150)		
Basic U-net	0.852 $\pm$ 0.078	< 0.001
Dense U-net	0.857 $\pm$ 0.079	< 0.001
Residual U-net	0.836 $\pm$ 0.082	< 0.001
Residual Dense U-net	**0.870 **$\pm$** 0.074**	**–**

Results are indicated as mean ± standard deviation, and the best performances are indicated in bold.

PV, pancreatic volume; DSC, dice similarity coefficient.

*We used a paired *t* test to compare the residual dense u-net with the corresponding network and used a significance level of 0.05.

**Figure 3:**
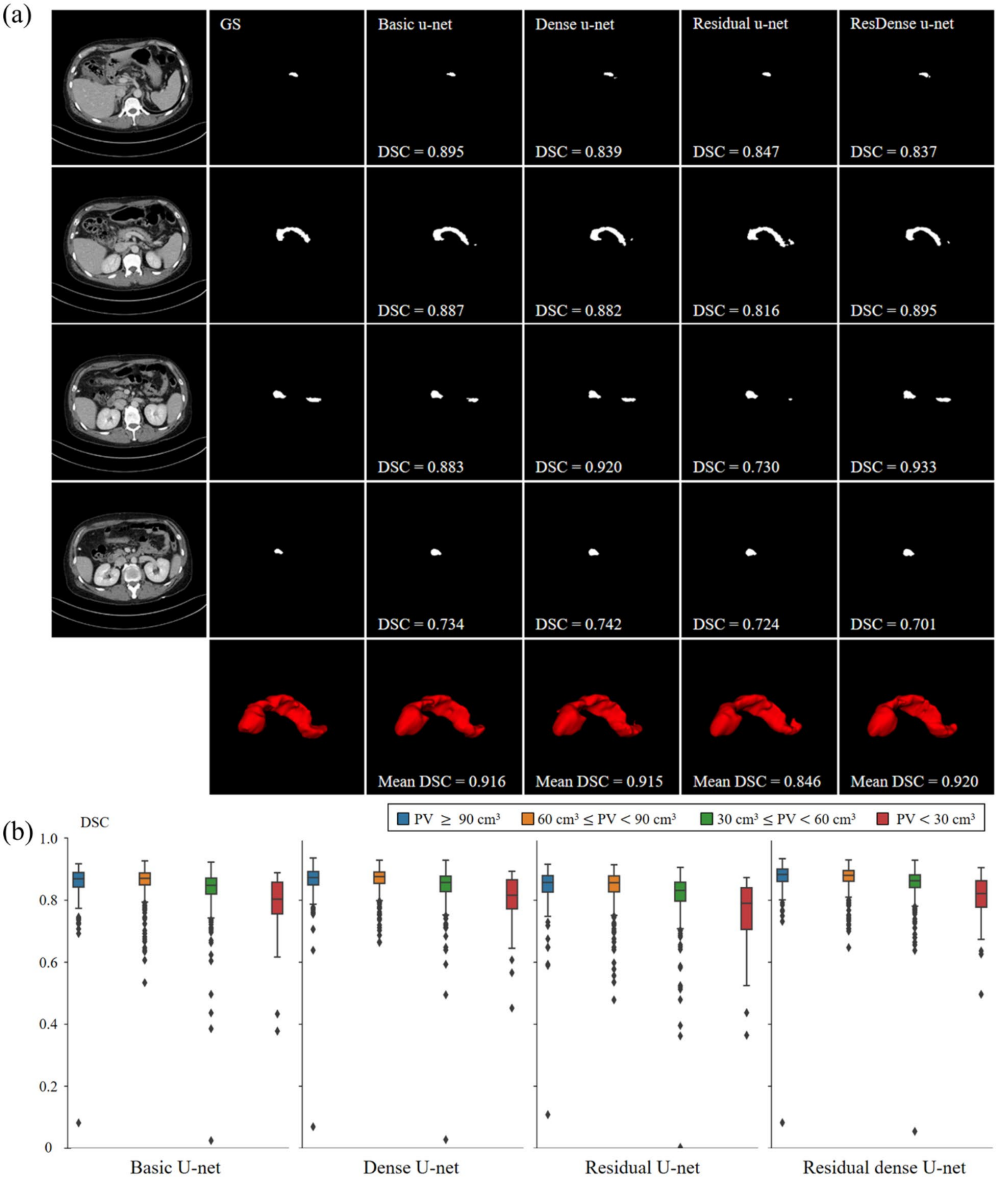
(**a**) Representative examples of pancreas segmentation in the 2D axial plane and 3D volume of one patient. (**b**) DSC metric in each deep learning model according to the volume of the pancreas. GS, gold standard; DSC, dice similarity coefficient; ResDense, residual dense; PV, pancreatic volume.

We visually assessed the four semantic segmentation models in the 2D axial plane and 3D volumes based on a single patient’s CT (Fig. 3). 3D visualization was conducted via 3D volume rendering with a 3D Slicer (ver. 4.11.20200930; http://www.slicer.org).

### Pancreas volume estimation

**Figure 4:**
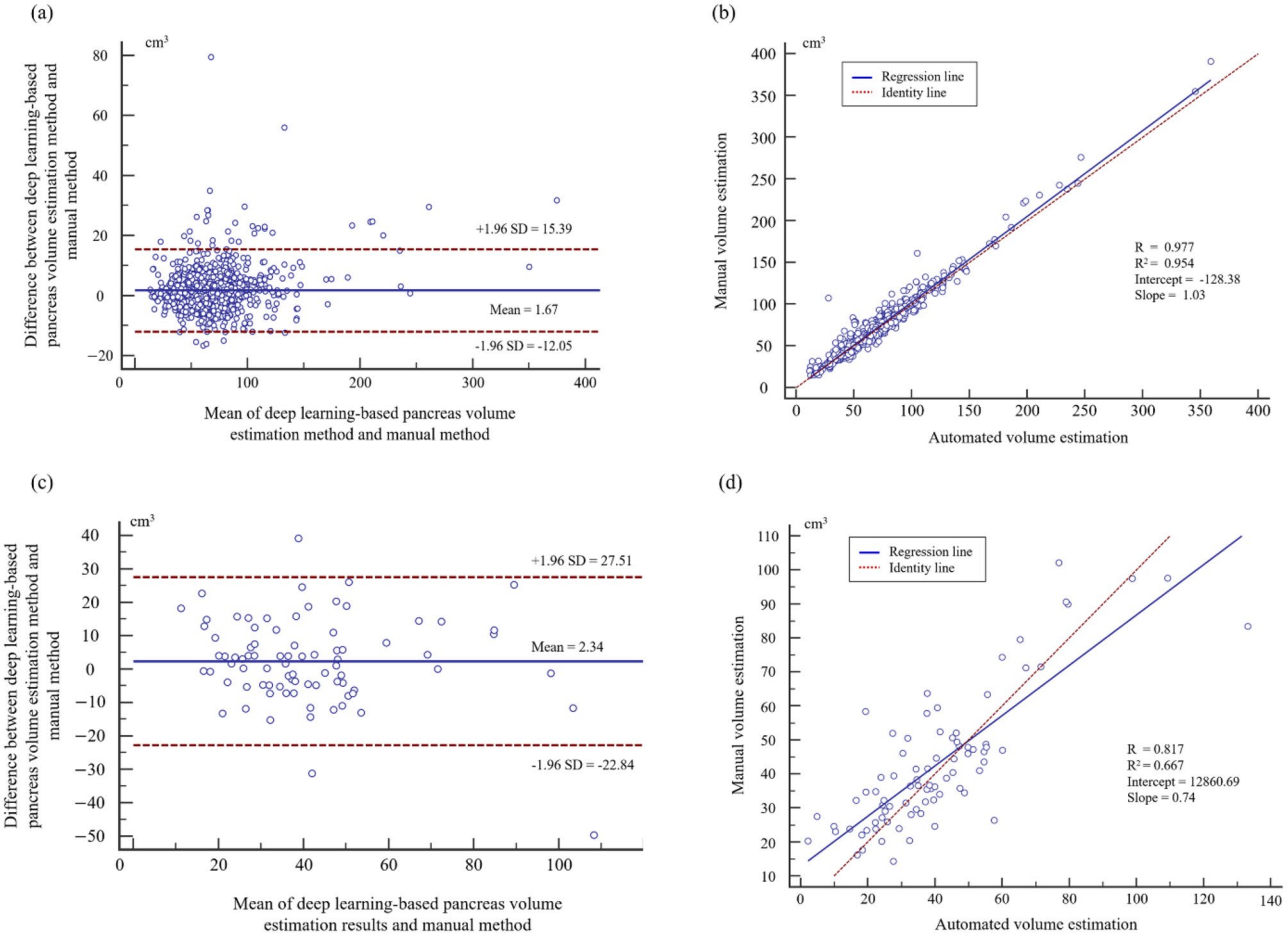
Estimation of pancreatic volume assessments using DL-prediction and manual pancreas segmentation. To validate the DL approaches, the (**a**) Bland–Altman plot and (**b**) regression plot were employed for internal validation and (**c**, **d**) external validation. SD, standard deviation.

We evaluated the pancreas volume estimation performance of the residual dense u-net using the Bland–Altman plot and regression analysis (Fig. 4). Most of the estimation errors outside the coefficient of repeatability (± 1.96 SD) were underestimated (n = 32). In contrast, over-estimations did not occur often (n = 4). We performed correlation and intraclass correlation coefficient (ICC) analyses for pancreatic volumetry. For the internal validation, we obtained an R^2^ score of 0.954 (*p* < 0.001) using the regression analysis, an R score of 0.977 (*p* < 0.001) using the correlation analysis, and an ICC score of 0.987. For the external validation, we obtained R^2^, R, and ICC scores of 0.667 (*p* < 0.001), 0.817 (*p* < 0.001), and 0.894, respectively. We used MedCalc Statistical Software (ver. 14.8.1, https://www.medcalc.org) for the statistical analysis.

## Discussion

This study presents an automated deep learning method for pancreatic segmentation and volumetry using the abdominal CT images of 1006 participants who underwent a health checkup. Recently, various studies have suggested a promising DL network for pancreas segmentation. However, to the best of our knowledge, there is no existing study on a DL approach applied and evaluated on a large abdominal CT dataset of more than 1000 patients. DL-based medical image segmentation is highly dependent on the number of data points. However, previously presented DL-based pancreatic segmentation studies used the NIH pancreas-CT dataset (n = 82). Although the previously proposed DL networks achieved high performance for pancreas segmentation (mean DSC of 0.866^11^, 0.854^13^ and 0.859^30^), there is insufficient data to prove that those networks are reliable. Therefore, in this study, we presented a DL-based pancreas segmentation on a large dataset (i.e., 1,006 abdominal CT images) and conducted external validation on the NIH pancreas-CT dataset using four state-of-the-art 3D segmentation networks. We demonstrated that residual dense u-net enables accurate pancreas segmentation and volumetry: (1) mean precision, recall, and DSC of 0.869, 0.842, and 0.842 for internal validation; (2) mean precision, recall, and DSC of 0.779, 0.749, and 0.735 for external validation. We confirmed that the number of trainable parameters is proportional to the segmentation performance of the DL approaches. The segmentation performance on the external NIH pancreas CT dataset was significantly inferior to that of the internal dataset. We assume that these results were attributable to the different slice thicknesses of the CT images; the external dataset was acquired using a 1.5–2.5 mm slice thickness.

In this study, a DSC comparison was performed according to four pancreatic volume (PV) ranges in four networks used for pancreas 3D-segmentation: (1) PV < 30 cm^3^, n = 54; (2) 30 cm^3^ ≤ PV < 60 cm^3^, n = 361; (3) 60 cm^3^ ≤ PV < 90 cm^3^, n = 441; (4) PV > 90 cm^3^, n = 150. In the total volume range, the residual dense u-net achieved the highest mean DSCs (Fig. 3b; Table 3). The mean DSC had a positive correlation with the pancreas volume, and all networks achieved the highest mean DSC results in samples with a volume of 60–90 cm^3^. Generally, the network achieves a high DSC, which is proportional to the volume of the pancreas. However, we assumed that high segmentation performance was achieved for samples with a pancreatic volume of 60–90 cm^3^ owing to the high ratio of 60–90 cm^3^ samples in the dataset (43.84%). In contrast, all the networks achieved the lowest segmentation performance for abdominal CT for patients with a pancreatic volume of 0–30 cm^3^.

We assessed the residual dense u-net-based pancreatic volume measurements using the Bland–Altman plot and regression plots (Fig. 4). The agreement between the network pancreatic volume measurement and the manual measurements was high, and there were mean differences between DL-based and manual-based pancreatic volume estimation. For the internal validation, the mean difference was 1.67 cm^3^, and the mean difference of the external dataset was 2.34 cm^3^. Most pancreatic volume estimation results were reliable; however, a few underestimations (n = 32) and over-estimations (n = 4) existed in a total of 1006 datapoints. Most of the underestimation occurred for a pancreatic volume greater than 90 cm^3^, which is presumed to be owing to the blurred boundary or low density of soft tissue.

This study presented a semi-automated pancreas segmentation approach based on DL methods for 1006 participants. However, there are several limitations to our study. We manually cropped the volume of interest (region of pancreas) to train the DL networks, owing to a lack of random access memory and GPU memory. Accordingly, further study is necessary to achieve fully automated pancreas segmentation using two-stage methods, such as cascaded or coarse-to-fine networks. Moreover, we assumed that other segmentation methods^31–34^ may be appropriate for accurate pancreas segmentation, owing to the blurry boundaries of the pancreas. The study of two-stage networks and a state-of-the-art segmentation strategy for more accurate pancreas segmentation will be part of our future work.

Repeatable and reproducible pancreas segmentation is necessary for pancreatic volumetry, and CNNs may have broad applicability to this problem. Furthermore, automated abdominal organ segmentation^35, 36^ and analysis applications can be used not only for CT but also for diverse modalities, such as magnetic resonance imaging and ultrasound. However, experiments using data that includes various races, ages, and pancreatic volumes are necessary to evaluate the applicability of these methods to clinical practice. Our study presented a DL-based semi-automated method on data from 1006 healthy Koreans; however, further DL-based studies on a dataset that includes various features are necessary to investigate reliable DL-based pancreas-segmentation strategies to aid clinicians.

## Data Availability

The datasets generated and/or analyzed during the current study are not publicly available because permission to share patient data was not granted by the institutional review board, but they are available from the corresponding author upon reasonable request.
